# Treatment trajectories of patients with borderline personality disorder prescribed pharmacotherapy: real-world insights from a retrospective observational study

**DOI:** 10.1186/s12888-026-07974-6

**Published:** 2026-03-20

**Authors:** Carissa White, Suzanne St.Rose, Jennifer B. Dwyer, Emily O. C. Palmer, Joannas Yeow, Kira Griffiths, Benjamin Chee, Mayowa Oyesanya, Rashmi Patel

**Affiliations:** 1 Boehringer Ingelheim Pharmaceuticals, Inc., Ridgefield, CT USA; 2 Boehringer Ingelheim International GmbH, Binger Strasse 173, 55216 Ingelheim am Rhein, Germany; 3 Holmusk Technologies, Inc., London, UK; 4 Holmusk Technologies, Inc., Singapore, Singapore; 5 Department of Psychiatry, University of Cambridge, Cambridge, UK

**Keywords:** Antidepressant, Borderline personality disorder, Mood stabilisers, Polypharmacy, Second-generation antipsychotics

## Abstract

**Background:**

Borderline personality disorder (BPD) is a significant cause of morbidity with no approved pharmacological treatment. We assessed treatment trajectories of patients with BPD in real-world clinical practice to help identify treatment gaps.

**Methods:**

This retrospective, observational, cohort study used de-identified MindLinc electronic health records data from the Holmusk NeuroBlu database (Version 21R2) to analyse the treatment journey of patients with BPD (aged ≥ 12 years with ≥1 diagnosis of BPD) that were prescribed pharmacological treatment within 14 days of diagnosis (baseline) and had treatment data for ≥12 months.

**Results:**

Of those prescribed pharmacological treatment at baseline, 1461 patients (16.1%) had 12 months of follow-up data. Antidepressants were the most frequently prescribed medication at baseline (80.4%) either alone or in combination with other medication classes, followed by second-generation antipsychotics (SGAs), anxiolytics and mood stabilisers. In the 12 months post-baseline, the most frequently recorded treatment pathway was a switch from 1 antidepressant to another. Sertraline (5.5%), fluoxetine (5%), and citalopram (5%) were the most-prescribed antidepressants; lamotrigine (24.9%), gabapentin (15.4%), and valproate (7.1%) were the most prescribed mood stabilisers; and quetiapine (22.1%) and aripiprazole (19.0%) were the most prescribed SGAs. Polypharmacy (defined as the prescription of ≥ 1 psychotropic medication) was observed in 83.1% of patients at baseline and increased with follow-up time and age.

**Conclusion:**

The high rates of polypharmacy observed suggest that current clinical practices may not fully align with treatment guidelines for BPD, and that patients with BPD experience a considerable treatment burden. Limitations of this study include the absence of psychotherapy data and the use of prescription records without information on treatment adherence. Nonetheless, the diversity of treatment patterns observed reflects the complex symptomatology of BPD and highlights the need to deepen our understanding of its neurobiology to improve pharmacological treatment strategies and translate to meaningful patient outcomes.

**Clinical trial number:**

Not applicable

**Supplementary information:**

The online version contains supplementary material available at 10.1186/s12888-026-07974-6.

## Background

Borderline personality disorder (BPD) is a complex psychiatric disorder that can manifest as instability in interpersonal relationships, self-image, affect, and behaviour. In the US, the estimated lifetime prevalence of BPD ranges from 1.4%–2.7% [1]. BPD is characterised by a high degree of impulsivity, fear of abandonment, intense anger, and suicidal thoughts/behaviours [2]. The onset of BPD is widely accepted to occur during adolescence with gradual progression through early adulthood [2, 3].

A substantial public health burden is attributed to BPD at a patient and societal level [4, 5]. Individuals diagnosed with BPD experience a lower quality of life and have a shorter life expectancy than those without [6, 7]. For example, one study found individuals diagnosed with a personality disorder to have a life expectancy at birth 17.7–18.7 years shorter than the general population in England and Wales [6]. BPD also imposes a considerable societal burden due to heavy utilisation of mental healthcare services and hospital facilities, work disability or the incapability to work, and the use of legal services [4, 8]. The annual direct healthcare costs and indirect costs through productivity losses in patients with BPD are estimated to exceed that of matched controls by 16-fold [9].

The burden of BPD is closely related to its complex symptomatology, which often makes diagnosis and subsequent treatment challenging [10]. Further, patients with BPD frequently present with high rates of comorbid mood, anxiety, substance use, and other personality disorders, that require clinical attention and drive acute treatment-seeking [11]. The US guidelines from the American Psychiatric Association (APA) published in 2024 recommend psychotherapy as a first-line treatment option for patients with BPD, which is linked to symptom-specific relief [12–15]. However, the evidence base for BPD-targeted psychotherapy is limited, particularly concerning its clinical benefit over standard treatment and the optimal time needed for sustained functional improvement [15]. There is currently no approved pharmacological treatment for BPD [16]; however, pharmacological treatment is often prescribed off-label, either alone, as an adjunct to psychotherapy, or for comorbid mental disorders [17, 18]. Although the APA guidelines acknowledge that pharmacological treatment has an important adjunctive role in some circumstances for BPD [15], UK guidelines from the National Institute for Health and Clinical Excellence (NICE) advise against pharmacological treatment except in cases of acute crisis [13, 19]. Studies have shown that second-generation antipsychotics (SGAs), anticonvulsants, and antidepressants do not consistently reduce the severity of BPD, although some symptoms such as anger, aggression, and affective lability may improve [20]. According to a recent meta-analysis, of the 87 commonly prescribed drugs for BPD, only 9 have been evaluated in randomised clinical trials [20]. Furthermore, few studies have investigated the use of psychotropic interventions for BPD in a real-world clinical setting. In a recent Italian study of 75 patients with a primary diagnosis of BPD, 98% were prescribed ≥ 1 medication, while most (82%) received 2 or more [21]. The authors noted that the presence of comorbid psychiatric symptoms rather than severity of BPD influenced prescribing [21].

Given the complex manifestations of BPD, polypharmacy is common, with patients typically being prescribed more pharmacological treatment than those with other psychiatric disorders [21]. However, there is a clear need for evidence-based treatment options for BPD due to the limited high-quality evidence supporting the efficacy of pharmacological treatment [22] and the aforementioned variation between national guidelines. Moreover, in the absence of large-scale observational studies on common treatment patterns in BPD, data derived from pre-existing electronic health records (EHR) can provide real-world evidence on current treatment trajectories in clinical practice. EHR-based analyses allow assessment of prescribing behaviours and polypharmacy over time, and can include large, diverse patient populations. These insights can help identify unmet needs and treatment gaps in real-world practice and therefore, inform future mental healthcare strategies for the management of BPD.

The objective of this study was to characterise the pharmacological treatment trajectory and frequency of polypharmacy in patients with BPD over the first 12 months following initial diagnosis. It was hypothesised that the medication class of treatments prescribed at baseline for patients would vary depending on the presence of comorbid symptoms, and that patients would experience switches between prescribed medication classes across the course of their treatment.

## Methods

### Data source

This retrospective, observational study utilised de-identified electronic health record (EHR) data from mental healthcare providers across 15 US states operating the MindLinc EHR system contained within the NeuroBlu database (Version 21R2) [23].

### Study design and setting

Patients were followed for ≥12 months after the index date, defined as the date of first recorded BPD diagnosis (International classification of disease [ICD]-9: 301.83/ICD-10: F60.3) in the NeuroBlu database. Due to variations in the frequency and/or timing of patient visits, a 14-day window on either side of the index date (baseline period), was applied to minimise the proportion of missing data and ensure adequate data capture (Supplementary Fig. 1).

### Participants

Eligible patients had ≥ 1 recorded diagnosis of BPD within the NeuroBlu dataset between 2001 and 2020, were aged ≥ 12 years at the time of diagnosis, had a prescription of ≥ 1 psychotropic treatment within the baseline period with a treatment minimum of 14 days, and ≥52 weeks of visit data from the index date. The requirement for only 1 recorded diagnosis of BPD for inclusion in the study cohort was based on the relative underdiagnosis of BPD in clinical practice as this would provide for a larger and therefore, better powered sample size. A minimum of 52 weeks of visit data from the index date was required, as this duration was considered adequate for a patient to interact with clinical services and to allow observation of treatment changes and trajectories in those prescribed psychotropic medications. The requirement for ≥1 psychotropic prescription within 14 days of diagnosis reflects the study’s focus on characterising real-world pharmacological treatment patterns among individuals receiving such interventions, rather than all individuals diagnosed with BPD, some of whom may not receive pharmacotherapy. Patients were excluded if they had a current diagnosis of paranoid, schizoid, schizotypal, and antisocial personality disorders or lifetime diagnosis of schizophrenia, schizoaffective disorder, schizophreniform disorder, bipolar I disorder or delusional disorder. These exclusion criteria were applied to align with those employed in a related Phase 2 clinical trial (NCT04566601) that was ongoing at the time of the present study [24]. In particular, excluding patients with a current or lifetime diagnosis of bipolar I disorder was intended to enhance internal validity, enabling a focus on treatment trajectories specific to BPD pathology; including individuals with bipolar disorder could have obscured patterns unique to BPD due to differing pharmacological regimens.

### Ethical considerations

This study was conducted in accordance with the 1964 Declaration of Helsinki and its subsequent amendments (October 2013). A waiver of HIPAA authorisation was obtained prior to study conduct and covers data originating from all sites represented. Approval was granted by the

Western-Copernicus Group (WCG) Institutional Review Board (The Holmusk Real-World Evidence Parent Protocol; IRB registration number 1-1470336-1; Protocol ID HolmuskRWE_1.0).

### Outcomes

Data extracted from the EHR database was comprised of structured patient-level data, including sociodemographic information (e.g., age), quantitatively measured clinical variables (e.g., number of prescribed medications) and unstructured free text from the mental state examination (MSE). Using a previously developed and validated Natural Language Processing (NLP), this unstructured free text was transformed into structured, quantifiable data [23, 25]. Performance characteristics and validation metrics of the NLP approach have been reported previously and are not re-evaluated in the present study [25]. Extracted symptom categories were operationally defined based on predefined clinical constructs (refer to Supplementary Table 1 for ‘MSE labels and categories associated with BPD symptoms’). Pharmacological prescription data were evaluated at baseline and for ≥12 months post index date. Where prescription data were for a longer period (e.g., up to 78 weeks), this information was also recorded.

Outcomes assessed included baseline demographic characteristics and pharmacological treatment in the overall population and by psychiatric comorbidity. Medication class-specific treatment journeys were assessed in the 12 months prior to baseline and from baseline to first change or 12 months. Frequency of polypharmacy at baseline and the 12-month follow-up period was also assessed. Polypharmacy was ascertained when multiple (>1) medications were prescribed within the same period for ≥14 days of treatment, to exclude pro re nata (PRN) or short-course medication. In cases where there were treatment switches within the time period, the maximum number of unique pharmacological treatments prescribed together were considered. A treatment change was defined as an initiation of a new medication regardless of whether the previous treatment was still being prescribed. Polypharmacy at baseline was stratified by psychiatric comorbidity and illness severity, defined by the Clinical Global Impression – Severity (CGI-S) scale as normal to mildly ill (score of 1–3), moderately to markedly ill (score of 4–5) or severely to most ill (score of 6–7).

To manage gaps in prescription data, when a patient was prescribed the same medication more than once, and this prescription occurred < 60 days from the end date of the previous prescription, the entire period was considered as a single treatment episode. This practice was applied to account for the discontinuity in follow-up visits in real-world clinical practice, which may involve non-attendance at scheduled visits where the clinician intended for the patient to remain on the same treatment. A feasibility assessment determined a length of 60 days between prescriptions of the same drug to constitute a single treatment episode. This management strategy helped to optimise the sample size for treatment episode data while minimising potential false positives (longer gap days) and false negatives (shorter gap days). Treatment continuation was evaluated as the proportion of time from the beginning to end of the prescription period that included a prescription of a particular medication class (treatment continuation percentage).

### Bias

As data from real-world clinical practice are not systematically recorded and records reflect patient interaction with healthcare providers, missing data and the distribution of EHR data might introduce selection bias. In the case of mental healthcare, patients who are more unwell may have increased contact with mental healthcare services and therefore have more data recorded and are more likely to meet inclusion criteria for entry into the study cohort. Conversely, patients who are more unwell or are experiencing poor social conditions, such as homelessness, may disengage from services and therefore have a higher proportion of missing data and are more likely to be underrepresented. To identify where results may be biased by confounding or other artefactual findings, data interpretation was conducted with input from clinical experts proficient in EHR data analytics.

### Statistical analysis

Treatment trajectories were visualised within each medication class. Sankey diagrams were generated to display population-level treatment pathways. For these diagrams, each pharmacological treatment was categorised to 1 medication class. See Supplementary Table 2 for categorisation. Descriptive statistics were used to examine pharmacological treatments and treatment combination data (proportions and frequencies).

## Results

### Baseline and demographics data

Of the 13,444 individuals aged ≥ 12 years with a diagnosis of BPD in the NeuroBlu database, 9221 did not have a lifetime/current diagnosis of certain comorbid psychiatric disorders (Supplementary Fig. 2). Of these, 1461 patients had 12 months of follow-up data and were receiving pharmacological treatment at baseline with duration of ≥14 days; this was the final analysis cohort for the study. The demographic characteristics of the full cohort of 13,444 individuals with BPD identified from the database and the cohort meeting the inclusion criteria for this study are presented in Table 1. Mean (SD) age at baseline of the analysis cohort was 35.2 (12.8) years and the majority (87.3%) of patients were female (Table 1). The most common comorbid psychiatric disorder was MDD (57.4%), followed by anxiety disorders (34.5%). At baseline, 1175 (80.4%) patients were first prescribed antidepressants either alone or in combination with other medication classes. The most common combinations were antidepressants with SGAs, anxiolytics or mood stabilisers.

**Table 1:** Demographic characteristics of the full BPD cohort from database and the cohort meeting the inclusion criteria for this study

Characteristic	Full cohort (n = 13,444)	Study cohort (n = 1461)
**Age at baseline (years)**		
Mean (SD)	33 (12.8)	35.2 (12.8)
**Age categories at baseline (years), n (%)**		
12–17	1084 (8.1)	87 (6.0)
18–25	3591 (26.7)	301 (20.6)
26–35	3654 (27.2)	415 (28.4)
36–45	2573 (19.1)	315 (21.6)
46–55	1789 (13.3)	247 (17.0)
56–65	617 (4.6)	82 (5.6)
>65	136 (1.0)	14 (1.0)
**Gender, n (%)**		
Female	11,241 (83.6)	1275 (87.3)
Male	2180 (16.2)	185 (12.7)
Unknown	23 (0.2)	1 (0.1)
**Race, n (%)**		
White	7940 (59.1)	876 (60.0)
Black or African American	1394 (10.4)	120 (8.2)
Others^a^	795 (5.9)	59 (4.0)
Unknown	3315 (24.7)	406 (27.8)
**Ethnicity, n (%)**		
Hispanic or Latino	1,412 (10.5)	204 (14.0)
Not Hispanic or Latino	5,402 (40.2)	677 (46.3)
Unknown	6,630 (49.3)	580 (39.7)
**Comorbidities at baseline, n (%)**		
MDD	5960 (45.7)	834 (57.1)
Other psychiatric disorders	4214 (32.3)	405 (27.7)
Anxiety disorders	3602 (27.6)	504 (34.5)
SUD	4514 (34.6)	449 (30.7)
PTSD	3804 (29.2)	476 (32.6)
Other bipolar disorders	3179 (24.4)	458 (31.4)
Other mood disorders	2318 (17.8)	306 (20.9)
Bipolar 1 disorder	2451 (18.8)	0 (0.0)
ADHD	849 (6.5)	130 (8.9)
Schizoaffective disorder	816 (6.3)	0 (0.0)
Eating-related disorders	663 (5.1)	101 (6.9)
No psychiatric comorbidities	332 (2.5)	0 (0.0)

^a^Includes Asian, Native American Indian and Pacific Islander

ADHD, attention deficit hyperactivity disorder; IQR, interquartile range; MDD, major depressive disorder; OCD, obsessive compulsive disorder; PTSD, post-traumatic stress disorder; SD, standard deviation; SUD, substance use disorder

### Treatment journeys 12 months prior to baseline

**Fig. 1:**
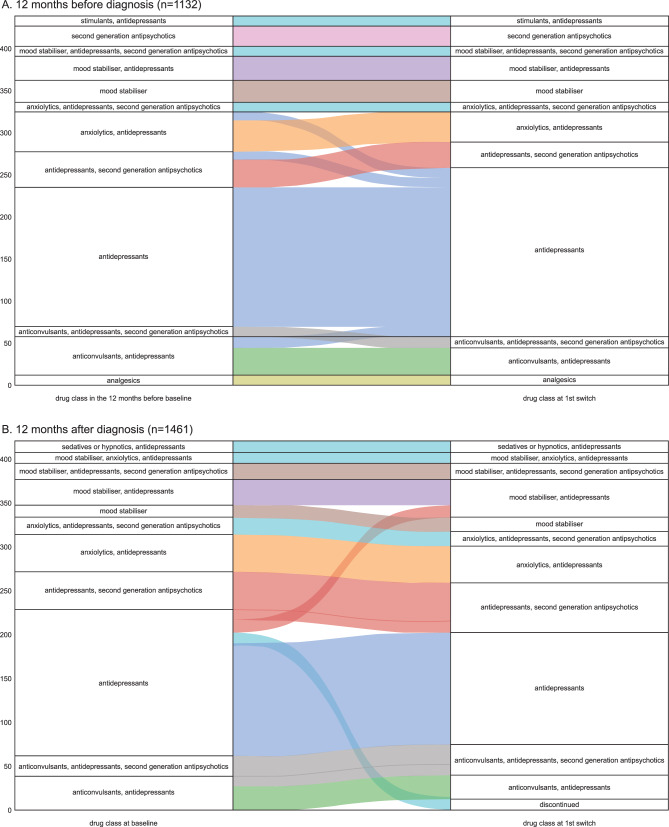
Sankey diagrams illustrating the most common first recorded treatment journey by medication class. The treatment trajectory patterns between each medication class are shown from left to right. The depth of the coloured lines is proportional to the number of patients as represented on the y-axis. Note: To improve interpretability, only the most common journeys are represented, so the patient count may not total the number of patients in sub-cohort A (12 months pre-baseline to 1^st^ treatment switch) and sub-cohort B (baseline to 1^st^ treatment switch)

Figure 1Aillustrates the treatment journey of patients with BPD (*n* = 1132 of the eligible 1461) across the 12 months prior to baseline. In the 12-month pre-baseline period, the majority of patients (*n* = 861, 76.1%) were prescribed antidepressants alone or in combination with other medication classes. A total of 33 (3.0%) patients discontinued use of the other medication classes and the prescription was switched to antidepressants. A small proportion of patients were prescribed SGAs or mood stabilisers and tended to maintain this prescription throughout the 12-month period. The 5 most common treatment journeys, as represented by the proportion of patients who continued each combination during the 12-months pre-baseline period, were antidepressants alone (14.8%), anxiolytics + antidepressants (3.2%) and antidepressants + anticonvulsants (2.8%) (Supplementary Table 3). Throughout the 12-month period, patients typically continued both with the medication class initially prescribed, and the individual medications prescribed within these classes (Supplementary Table 4). The most frequently prescribed antidepressants in the 12-month period before baseline were fluoxetine (8.4%), sertraline (6.9%), and citalopram (6.5%). The number of patients prescribed a first-generation antipsychotic in the year prior to baseline was very low (*n* = 17). It was therefore not possible to make any inference about common treatment trajectories of first-generation antipsychotics.

### Treatment journeys 12 months after baseline

The 5 most common treatment journeys for patients with BPD 12 months after baseline are shown in Fig. 1B and Supplementary Table 3. The most frequent treatment journeys were antidepressants alone (8.8%), SGAs + antidepressants (3%) and anxiolytics + antidepressants (2.9%). The most frequently prescribed antidepressants were sertraline (5.5%), fluoxetine (5%), and citalopram (5%). The most frequently prescribed SGAs were quetiapine (22.1%) and aripiprazole (19.0%); patients tended to either persist with these drugs or discontinue SGAs altogether.

### Polypharmacy

**Fig. 2:**
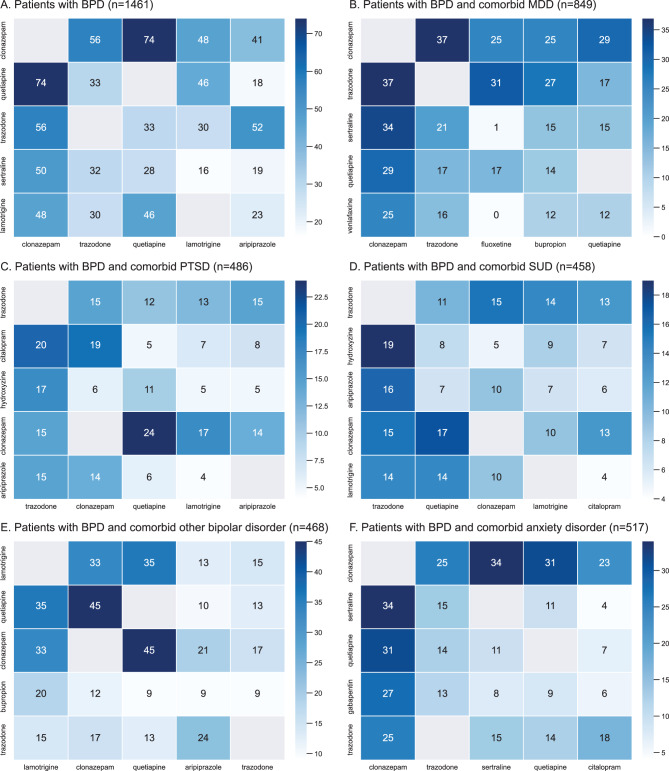
Heatmaps illustrating treatment combinations among patients with BPD who were prescribed more than 1 medication. BPD, borderline personality disorder; PTSD, post-traumatic stress disorder; MDD, major depressive disorder; SUD, substance use disorder. Number in each cell: number of patients. Dark blue cell denotes a higher number of patients with a treatment combination; light blue denotes a lower number of patients with a treatment combination; blank cell denotes no patients with the corresponding treatment combination. Note: a patient may be present in more than 1 combination category

Polypharmacy was observed in most patients, with 83.1% of the study population being prescribed ≥ 1 medication at baseline (Table 2). Further, polypharmacy rates increased at 18 months after baseline, with >90% of patients being prescribed ≥ 1 pharmacological treatment. The most common treatment combination across all patients with BPD was clonazepam and quetiapine (*n* = 74; 5.1%) (Fig. 2A). However, none of the combinations were particularly common, suggesting a high degree of variability in the prescription of medication combinations at baseline. Supplementary Table 5 shows the most frequently prescribed pharmacological treatments at baseline stratified by age group. Despite small sample sizes in some age groups, the proportion of patients with BPD who were prescribed ≥ 1 medication at baseline appears to increase with age until 46–55 years, after which polypharmacy rates tended to decrease.

**Table 2:** Unique medications prescribed to patients with BPD at baseline and ≤18 months post baseline

Number of unique medications prescribed, n (%)	Baseline (n = 1461)	10 weeks (n = 1228)	6 months (n = 1043)	12 months (n = 949)	18 months (n = 693)
1	247 (16.9)	172 (14.0)	115 (11.0)	95 (10.0)	59 (8.5)
2	361 (24.7)	256 (20.8)	213 (20.4)	184 (19.4)	143 (20.6)
3	296 (20.3)	289 (23.5)	253 (24.3)	193 (20.3)	123 (17.7)
4	247 (16.9)	226 (18.4)	177 (17.0)	203 (21.4)	142 (20.5)
>4	310 (21.2)	285 (23.2)	285 (27.3)	274 (28.9)	226 (32.6)
**Number of patients with** $>$ **1 medication prescribed, n (%** **[95% CI])**	1214 (83.1 [81.2–85.0])	1056 (86.0 [84.1–87.9])	928 (89.0 [87.1–90.9])	854 (90.0 [88.1–91.9])	634 (91.5 [89.4–93.6])

BPD, borderline personality disorder; CI, confidence intervals

Polypharmacy was observed among most patients with BPD and comorbid psychiatric disorders at baseline (78.4%–91.2%) (Supplementary Table 6). Patients with other comorbid mood disorders had the lowest proportion of polypharmacy at baseline (78.4%), while those with obsessive compulsive disorder (OCD) had the highest proportion of polypharmacy (91.2%). Although not frequently prescribed, the most commonly occurring combinations across all patients with a comorbid psychiatric disorder were clonazepam + quetiapine, clonazepam + trazodone, amphetamine + dextroamphetamine or clonazepam + sertraline (Fig 2B–F). The most common treatment combination for patients with MDD as a comorbidity was clonazepam + trazodone (*n* = 37; 4.4%), post-traumatic stress disorder as a comorbidity was clonazepam + quetiapine (*n* = 24; 4.9%), substance use disorders (SUD) as a comorbidity was hydroxyzine + trazodone (*n* = 19; 4.1%), other bipolar disorder was clonazepam + quetiapine (*n* = 45; 9.6%), and anxiety disorder as a comorbidity was clonazepam + sertraline (*n* = 34; 6.6%).

Baseline polypharmacy rates by illness severity were highest in patients classified by CGI-S as ‘severely to most ill’ (*n* = 161, 89.4%), followed by ‘normal to mildly ill’ patients (*n* = 134, 87.6%) and ‘moderately to markedly ill’ patients (*n* = 873, 81.7%).

Patients with 12 months of follow-up data were evaluated for the proportion of time that they were prescribed medications. Antidepressants were continued for the longest proportion of time (81.8% [95% confidence intervals, CI: 80.3–83.3] of the 12-month period) compared with other medication classes (49.8% [95% CI: 41.6–58.0] to 71.0% [95% CI: 66.7–75.3]) (Table 3). When stratified by symptom class as documented in the MSE, patients with symptoms of suicidal attempt/self-injury received the longest continued prescription of antidepressants (84.0% [27.2%]), SGAs (74.8% [30.5%]), stimulants (77.9% [32.9%]), and SUD drugs (70.5% [37.7%]). Conversely, these patients had the shortest prescription of hypnotics or sedatives (Table 3).

**Table 3:** Mean treatment continuation of patients with BPD (*n* = 1461) stratified by symptom class

	Medication class									
	Anti- depressant	FGA	SGA	Anti-convulsant	Anxiolytics	Hypnotics and sedatives	Stimulants	Analgesics	SUD drugs	Mood Stabiliser
**Number of patients with ≥12 months of treatment data, n (%)**	1287 (88.1)	68 (4.7)	775 (53.0)	511 (35.0)	593 (40.6)	316 (21.6)	229 (15.7)	242 (16.6)	123 (8.4)	619 (47.0)
**Mean treatment continuation percentage, % [95% CI] (SD)**	81.8 [80.3–83.3] (26.6)	49.8 [41.6–58.0] (34.3)	68.7 [66.4–71.0] (32.1)	68.6 [65.7–71.5] (33.6)	63.4 [60.7–66.1] (33.4)	63.1 [59.3–66.9] (34.3)	65.2 [60.8–69.6] (33.9)	71.0 [66.7–75.3] (33.9)	63.6 [57.5–69.7] (34.5)	69.4 [66.8–72.0] (33.1)
**Symptom class, % (SD)**										
Impulsivity	100.0 (0.0)	-	100.0 (0.0)	-	-	-	-	100.0 (0.0)	100.0 (0.0)	-
Emotional dysregulation	78.7 (28.9)	43.7 (36.3)	66.9 (33.7)	69.4 (34.2)	63.3 (33.4)	62.3 (35.5)	70.5 (30.9)	72.7 (33.9)	59.9 (32.7)	68.5 (35.0)
Suicidal intent/ideation	82.4 (27.5)	35.2 (33.5)	72.6 (31.6)	64.6 (35.2)	62.4 (33.6)	57.9 (33.0)	63.0 (34.3)	66.8 (35.6)	65.9 (37.2)	69.0 (34.3)
Suicidal attempt/self-injury	84.0 (27.2)	31.6 (32.8)	74.8 (30.5)	64.6 (34.8)	62.7 (33.1)	51.5 (32.8)	77.9 (32.9)	65.3 (35.7)	70.5 (37.7)	67.8 (34.8)

BPD, borderline personality disorder; CI, confidence intervals; FGA, first-generation antipsychotic; SD, standard deviation; SGA, second-generation antipsychotic; SUD, substance use disorders

## Discussion

Across the treatment journeys of the study cohort, antidepressants were frequently prescribed alone or in combination with other medication classes, with prescription rates of 76.1% in the 12-month pre-baseline period and 80.4% at baseline. Similarly, the prescription of antidepressants either alone or in combination with other psychiatric mediation was reported in an Italian study in which almost two-thirds of 75 patients across 5 clinical settings were prescribed ≥ 1 antidepressant [21]. However, the study noted that the prescription rates for benzodiazepines and antipsychotics were higher than antidepressants, substantiating the reported decline in antidepressant use in patients with BPD and the growing use of mood stabilisers and SGAs [26]. Furthermore, a recent 10-year analysis in Sweden reported a significant decline in antidepressant prescriptions for individuals with personality disorders, with and without comorbid psychiatric disorders [27]. The discrepancy in the findings of the present study may reflect the historical nature of the NeuroBlu database at the time the study was conducted and potential intercountry variation in prescribing practices.

While no medication combination was particularly common across all patients with BPD with and without comorbidities in this study, clonazepam + quetiapine was the most frequent combination reported. The frequency of quetiapine aligns with previous studies examining prescribing patterns in BPD [28–30] and is an expected finding given its known mood-stabilising, sedating and antidepressant effects depending on dose range [31]. At lower doses quetiapine has a strong antihistaminergic and therefore sedating effect, whereas at intermediate and higher doses, quetiapine can exert mood-stabilising and antidepressant and anxiolytic effects [31]. Meanwhile, the finding for clonazepam is less expected as current clinical guidelines generally advise against prolonged benzodiazepine use due to the risk of dependency and misuse [1]. However, earlier guidance in the APA 2001 BPD practice guidelines recommended adjunctive use of clonazepam for patients with BPD with affective dysregulation manifesting as anxiety [32]. This historical context may explain the observed prescribing patterns in the present study, as the NeuroBlu dataset includes patient records from 2001 to 2020.

Within medication classes, patients who were prescribed the most common antidepressants were found to generally persist with the prescription for the full 12-month follow-up period. Indeed, antidepressants were prescribed for an average 81.8% of the 12-month period. In the cases of SGAs and mood stabilisers, patients were also often prescribed the medications for the full follow-up period or discontinued the treatment class altogether. This pattern may be partially related to the typical length of prescription associated with the different medication classes. For example, SGAs are generally recommended to be used on a short-term or PRN basis for BPD [33]. On the other hand, antidepressants are generally used to address persistent comorbid depressive or anxiety symptoms in BPD [33]. Additionally, antidepressants take several weeks to demonstrate clinical effect and when they are well established, a gradual withdrawal of the medication is required [33]. Furthermore, most antidepressants are reasonably well tolerated, particularly selective serotonin reuptake inhibitors (SSRIs), which may reduce the likelihood of medication cessation due to tolerability problems. Together, these factors may contribute to the extended continuation of antidepressants compared to the other classes of medications assessed.

Polypharmacy, defined here as being prescribed more than 1 psychotropic medication, was reported for most patients in the study population despite the current absence of an approved pharmacotherapy for BPD. This finding aligns with previous studies in which high levels of polypharmacy were reported [16, 28, 29, 34, 35]. A large-scale cross-sectional survey conducted in the UK found that 48% of patients with BPD received ≥ 2 psychotropic medications [34]. Another study assessing the prescriptions of 2195 in-patients with BPD found that 80% of patients received ≥ 2 medications, and 54% of patients received ≥ 3 [29]. A US study of 290 patients with BPD found similar results, with 65.5% of patients receiving multiple psychotropic medications and 45.5% receiving intensive polypharmacy, defined as ≥ 3 standing medications at the same time [35]. A recent study using national databases in New Zealand found that 50% of people with BPD were prescribed ≥ 3 psychotropic medications in 2014, increasing to 55.9% in 2019 [28]. While polypharmacy is widely reported in individuals with BPD, there is a clear lack of consensus concerning its definition. A multitude of polypharmacy definitions are present in existing literature, with Masnoon et al. identifying over 100 definitions in their systematic review [36]. This extensive heterogeneity hinders the comparability of findings between studies and compromises current understanding of the treatment landscape in BPD, necessitating a standardised definition for polypharmacy. Nonetheless, the high rates of polypharmacy observed suggest that a high symptom burden may remain following treatment, which highlights the need for consensus guidelines and high-quality evidence substantiating the efficacy of pharmacological treatment in BPD management.

Our results revealed that polypharmacy rates increased at 18 months post-first recorded diagnosis, with over 90% of patients prescribed ≥ 1 medication. This finding contrasts that of a prospective US study spanning 16 years in which the prescription of ≥ 2 medications was found to decline from baseline to 8-year follow-up [37]. However, a cross-sectional study examining EHRs of individuals with personality disorders found polypharmacy, defined as ≥ 3 prescribed medications, to remain prevalent over a 10-year period with a trend towards reduced polypharmacy among patients without comorbid psychiatric conditions [27]. This pattern may reflect adherence to guidelines recommending regular medication reviews to establish whether treatment should be tapered or discontinued once symptoms have stabilised [1]. The discrepancy in polypharmacy rates between the present study and previous research may reflect differences in the timeframes analysed (8 years vs 18 months), the average age of the study cohorts (younger vs older patients), or the varying definitions of polypharmacy used. Age-related differences in prescribing patterns of polypharmacy may also reflect generational differences in prescribing practices among physicians. For example, in the prospective study, older patients may have been treated for BPD when younger and maintained on a medication regime which may be outdated by the standards of modern clinical practice [37]. Indeed, the prospective study only evaluated patients with BPD aged between 18-35 years. Our results also revealed that patients between the ages of 12–17 years were the least likely to receive multiple medications [37]. However, it should be noted that clinical guidelines recommend against the use of pharmacological treatment for BPD in adolescents [38].

Polypharmacy was observed for most patients with BPD and comorbid psychiatric disorders. Among those with comorbidities related to anxiety or depression, treatment combinations containing antidepressants were more common, which is in accordance with APA clinical guidelines for the treatment of BPD with comorbid psychiatric disorders [1]. Interestingly, the finding that clonazepam + trazodone was the most common medication combination in patients with comorbid MDD is somewhat unexpected given that SSRIs are the typical first-line treatment for MDD [1]. As previously noted, earlier APA guidelines recommend clonazepam as adjunctive treatment for BPD with anxiety symptoms, which may account for its use in patients with BPD and comorbid MDD [39]. In addition, trazodone possesses sedative properties and may be prescribed off-label to achieve the clinical effect of other medication classes, such as benzodiazepines [40]. Unlike benzodiazepines and other sedative and anxiolytic medications, trazodone is not a controlled substance [40]. Therefore, clinicians may be more inclined to prescribe trazodone to patients due to a perceived lower risk of misuse and dependence, which may account for the observed prescription rates in patients with BPD and MDD. It should however be noted that once the cohort was stratified by comorbid conditions in our analysis, the sample sizes became small, so the results should be interpreted with caution. In addition, careful consideration should be given to the prevalence of comorbid psychiatric disorders, which may share overlapping symptoms with BPD, such as impulsivity and mood dysregulation [1]. Given the current study did not evaluate symptom history, it is possible that comorbid psychiatric conditions were overdiagnosed among the study cohort, and thus overtreated.

While patients classed as ‘severely to most ill’ had higher rates of polypharmacy at baseline than those classed ‘moderately to markedly ill’ or ‘normal to mildly ill’, there was not a substantial difference observed by illness severity. This finding may reflect the fact that patients classified as ‘moderately to markedly ill’ constituted over three-quarters of the study cohort, which may have led to underestimation of the association between illness severity and polypharmacy. Similarly, a study in Italy of 75 patients with BPD found no significant associations between binary illness severity (more severe vs less severe) and pharmacological treatment by medication class [21]. Future investigations could enhance current knowledge by monitoring illness severity throughout follow-up periods to capture longitudinal trends in polypharmacy rates, and assess illness severity on a continuous scale to detect more granular changes.

Symptoms of suicidal attempt/self-injury as identified in the MSE were observed to receive the longest continuation of prescriptions of antidepressants, SGAs, stimulants and SUD drugs, likely reflecting symptom severity. This is consistent with the reported use of medications such as quetiapine and benzodiazepines to address symptoms of suicidal ideation or self-harm [18]. A recent review also suggests that stimulants, particularly methylphenidate, may be associated with improvements in impulsivity and related outcomes among individuals with BPD and comorbid ADHD, and could potentially lower the risk of suicidal behaviours. However, the current evidence is limited and largely observational, highlighting the need for more robust research through larger controlled trials focused on patients diagnosed primarily with BPD [41]. Notably, these prescribing patterns do not appear to align with APA guidelines, which recommend pharmacotherapy to be used only on a time-limited basis to reduce the short-term risk of self-harm in BPD [1]. Furthermore, clinical guidelines advise against prolonged use of benzodiazepines given their associated risk of dependency and misuse [1]. This is a particular concern for individuals with comorbid SUD, one of the most common psychiatric comorbidities associated with BPD, and further highlights the need for additional research examining symptom-specific prescribing patterns [11].

The present study has several strengths. Firstly, the longitudinal nature of this analysis allowed patient-level trends to be examined. Secondly, the NeuroBlu dataset contains a large cohort of patients with BPD receiving mental healthcare across 15 states within the US [23] and is therefore, likely to represent routine US prescribing patterns. Thirdly, the present study employed stringent inclusion criteria of patients who were prescribed medication at baseline and had 12 months of follow-up data, which enabled analysis from a uniform cohort and minimised complications due to missing data. A key limitation of this study is the lack of data on psychotherapy use among this cohort. Patients may have been concurrently receiving psychotherapy, as recommended by APA clinical guidelines, which should be considered when interpreting our findings [1]. Furthermore, there were some limitations associated with the inclusion criteria, such as the requirement of ≥ 1 psychotropic treatment at baseline, which may overestimate the prevalence of pharmacological treatment patterns relative to the overall BPD population and thus limit external validity. Restricting the cohort to patients who were prescribed a psychotropic medication within 14 days of diagnosis and had ≥ 12 months of follow-up data likely resulted in a sample that is more clinically severe, more engaged with services, and more likely to receive pharmacological treatment than the broader BPD population. In addition, excluding patients with a wide range of comorbid psychiatric disorders including schizophrenia spectrum disorders, delusional disorder, and bipolar I disorder may have led to a sample that is less clinically complex or severe compared to the overall BPD population, as individuals with multiple comorbidities often experience greater illness burden. Also, relying on only one recorded diagnosis of BPD may have increased the risk of misclassification due to challenges in differentiating BPD from other psychiatric conditions such as other personality disorders or adult ADHD in real-world settings [42–44]. Moreover, the definition of polypharmacy, which required a minimum 14-day overlap of prescribed medications, may have resulted in misclassifying patients undergoing medication switches as experiencing polypharmacy. As a result, the generalizability of the study findings may be limited and should be considered when interpreting the results. The exclusion of patients with comorbid bipolar I disorder further limits generalizability, as individuals with both conditions are known to be more heavily medicated and may follow different treatment pathways than those with BPD alone. Additionally, although the current analysis required that each medication be classified into a single medication class, some medications can be used for multiple indications dependent on dose or modality. For example, benzodiazepines can be characterised as anti-convulsants or anxiolytics, and gabapentinoids as anti-convulsant, analgesic or anxiolytic, depending on the indication [45]. When considering that gabapentin, a gabapentinoid, is among the top 5 individual medications prescribed in the US, this inconsistent classification may overstate findings by medication class [46]. A further limitation of this analysis is that the absence of clinical history data prior to entering the EHR limited the analysis to the first recorded treatment after diagnosis, rather than the first-line treatment. Data concerning the indication for which medications were prescribed were unavailable in the NeuroBlu dataset, which complicates the analysis of treatment pathways given the high frequency of comorbid conditions in BPD. Moreover, since adherence data for the medications prescribed was unavailable, the core focus of this analysis was on the treatments prescribed rather than taken. The small sample size of subgroups, as well as the exclusive focus on US data, are further limitations that may compromise the external validity of findings.

## Conclusions

The analysis found that antidepressants, SGAs, and mood stabilisers were the most frequently prescribed medications for patients with BPD. There was a difference in the prescription of common medication combinations depending on the presence of comorbidities. We also found that polypharmacy was common and increased with follow-up time and age. Overall, our findings of high rates of polypharmacy suggest that current clinical practices are not completely in alignment with current treatment recommendations for BPD, and that patients with BPD endure a considerable treatment burden. Further research on the treatment patterns of patients with BPD, including how these patterns have changed over time and differences at the individual medication level, will help inform prescription decisions, improve treatment outcomes, and identify areas of unmet need for novel therapeutic development. Additionally, future studies should consider examining treatment stability, as understanding the proportion of patients with stable versus unstable pharmacological regimens could help clarify the extent to which current management strategies are effective in maintaining symptom control and reducing treatment burden for patients with BPD. It would also be valuable for future research to compare patient characteristics and outcomes between those receiving specific medications to provide further insight into real-world management of BPD.

## Electronic supplementary material

Below is the link to the electronic supplementary material.

Supplementary Material 1

## Data Availability

To ensure independent interpretation of observational study results and enable authors to fulfil their role and obligations under the ICMJE criteria, Boehringer Ingelheim grants all external authors access to observational study data pertinent to the development of the publication. In adherence with the Boehringer Ingelheim Policy on Transparency and Publication of Clinical Study Data, scientific and medical researchers can request access to clinical study data when it becomes available on https://vivli.org/, and earliest after publication of the primary manuscript in a peer-reviewed journal, regulatory activities are complete, and other criteria are met. Please visit https://www.clinicalstudies.boehringer-ingelheim.com/msw/datatransparency for further information. The data supporting this study originate with Holmusk Technologies, Inc. These de-identified data may be made available upon request and are subject to license agreement with Holmusk. Interested parties should contact [publications@holmusk.com](mailto:publications@holmusk.com) to determine licensing terms.

## Abbreviations

APA: American Psychiatric Association
BPD: Borderline personality disorder
HER: Electronic health record
HIPAA: Health insurance portability and accountability act
ICD: International classification of disease
MSE: Mental state examination
NICE: National Institute for Health and Clinical Excellence
SGA: Second-generation antipsychotics
SUD: Substance use disorders
UK: United Kingdom
US: United States
