# Supplementary material for: Treatment trajectories of patients with borderline personality disorder prescribed pharmacotherapy: real-world insights from a retrospective observational study
Source: BMC Psychiatry. 2026 Mar 20;26:351. doi: 10.1186/s12888-026-07974-6 (PMC13127037; doi:10.1186/s12888-026-07974-6)
Supplement: Supplementary file 1 — Supplementary Material 1 [file 12888_2026_7974_MOESM1_ESM.docx]

## Supplementary material

### ****Supplementary Figure 1: Study design**
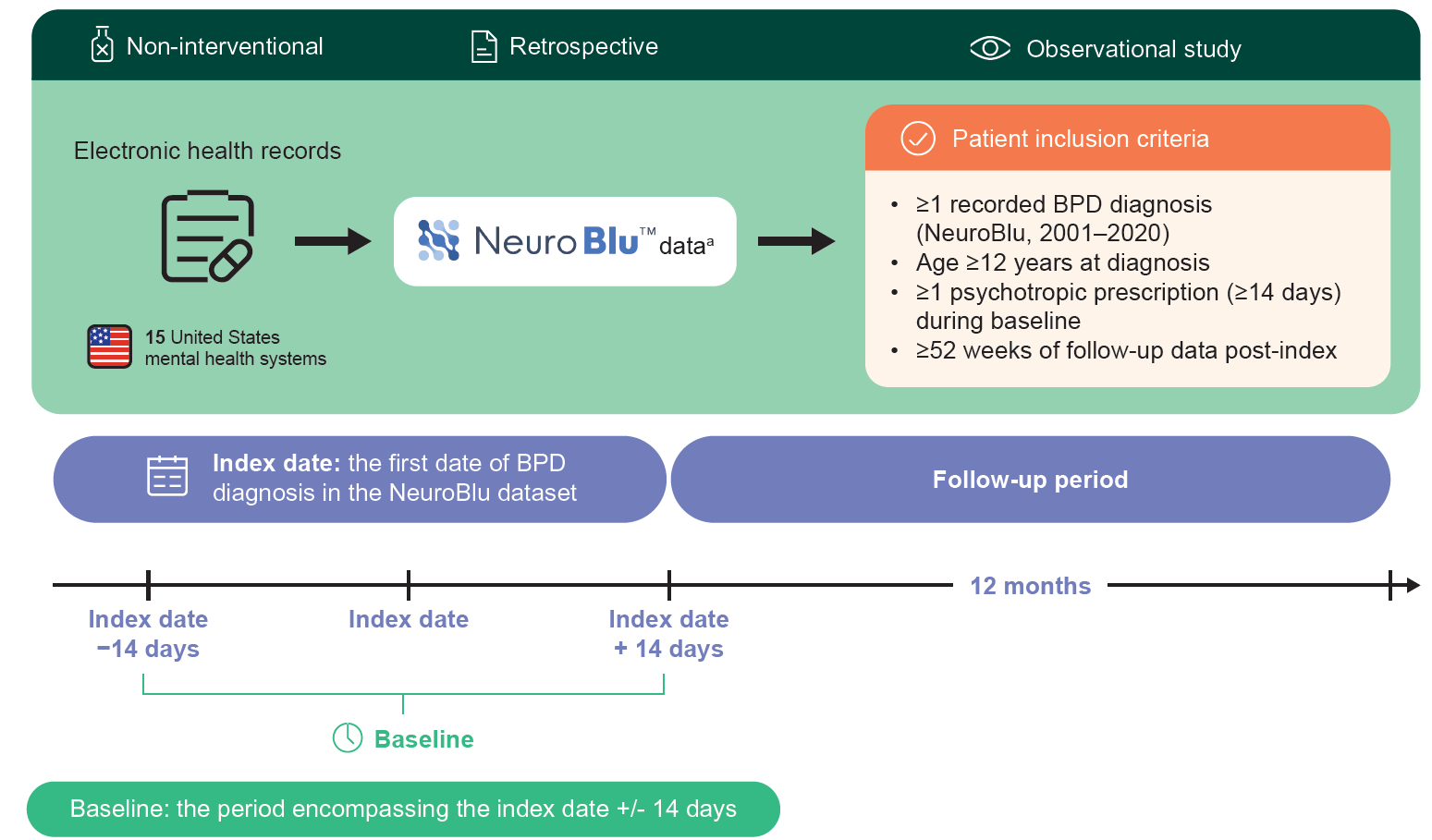
** ^a^A longitudinal behavioural health real-world database comprising both structured and unstructured patient-level clinical data.

### ****Supplementary Figure 2: Patient flow chart****


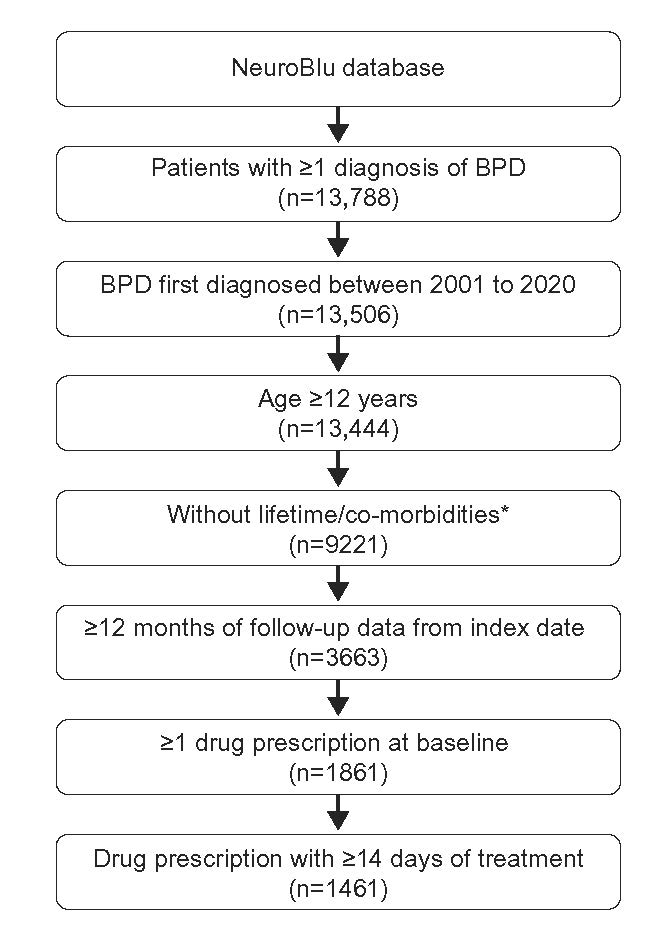


*Current diagnosis of paranoid, schizoid, schizotypal and antisocial personality disorders. Lifetime

diagnosis of schizophrenia, schizoaffective disorder, schizophreniform disorder, bipolar I

disorder, or delusional disorder.

BPD, borderline personality disorder.

### Supplementary Table 1: MSE labels and categories associated with BPD symptoms

| **MSE category** | **MSE factors** | **Sample text snippets** |
| --- | --- | --- |
| **BPD symptom category: Impulsivity** | | |
| Impulse control | Limited/some issues | “limited, but likely improved” |
|  | Poor/serious issues | “poor impulse control during depressed mood”, “history of poor impulse control” |
| **BPD symptom category: Emotional dysregulation** | | |
| Affect | Aggressive | “irrate, hostile, demanding, threatening suicidal ideation / homicidal ideation”, “easily angered and enraged” |
|  | Irritable/angry | “angry at first, more euthymic as session progressed”, “argumentative/testy”, “at times irritable” |
|  | Labile | “fluctuates from smiling to tearful periods per subject”, “initially calm and cooperative, as discussion of symptoms progressed became increasingly agitated” |
|  | Intense | “intense stare at times”, “glares at times”, “demanding” |
| Mood | Irritable, angry | “annoyed”, “constricted”, ”hostile”, ”resentful” |
|  | Labile | “labile”, “up and down” |
| **BPD symptom category: Suicidal intent / ideation** | | |
| Mood | Suicidal | “I want to kill myself” |
| Suicidality | Suicidal ideation | “chronic suicidal thoughts without intent”, “threats of suicide”, “verbally contracts for safety” |
|  | Suicidal with intent | “suicidal ideation with plan & intent” |
|  | Suicidal with plan | “suicide ideation with plan but no current intent” |
|  | History of ideation | “history of recent suicidal statements”, “denied current suicidal ideation, has recent history at intake” |
|  | Suicidal ideation with means | “with means” |
| **BPD symptom category: Suicidal attempt / self-injury** | | |
| Suicidality | Suicide attempt | “recent suicide attempt”, “current suicide attempt” |
|  | History of attempt | “history of suicidal overdose last attempt 2011”, “history of multiple suicide attempts” |
|  | Self-injurious | “self-mutilation” |
|  | History of self-injury | “history of self-injurious behaviors” |

### Supplementary Table 2: Pharmacological treatment class groupings of prescribed medications

| **Category** | **Medication compound** |
| --- | --- |
| Antidepressants | Amitriptyline, amoxapine, bupropion, butriptyline, citalopram, clomipramine, desipramine, desvenlafaxine, dothiepin, doxepin, duloxetine, escitalopram, esketamine, fluoxetine, fluvoxamine, imipramine, isocarboxazid, ketamine, levomilnacipran, maprotiline, melitracen, mianserin, milnacipran, mirtazapine, nefazodone, nortriptyline, paroxetine, phenelzine, protriptyline, reboxetine, sertraline, tranylcypromine, trazodone, trimipramine, tryptophan, venlafaxine, vilazodone, vortioxetine |
| Antipsychotics | 1^st^ Generation: chlorpromazine, droperidol, fluphenazine, haloperidol, loxapine, mesoridazine, molindone, perphenazine, pimozide, prochlorperazine, thioridazine, thiothixene, trifluoperazine  2^nd^ Generation: aripiprazole, aripiprazole lauroxil, asenapine, brexpiprazole, cariprazine, clozapine, Iloperidone, lumateperone, lurasidone, olanzapine, paliperidone, quetiapine, risperidone, ziprasidone |
| Analgesics | Acetaminophen, almotriptan, antipyrine, aspirin, butorphanol, diflunisal, dihydrocodeine, dihydroergotamine, eletriptan, ergotamine, fentanyl, frovatriptan, hydromorphone, levorphanol, meperidine, methoxyflurane, morphine, naratriptan, oxycodone, oxymorphone, pentazocine, propoxyphene, rizatriptan, salsalate, salicylamide, sumatriptan, tapentadol, tramadol, zolmitriptan |
| Mood stabilisers | Carbamazepine, lamotrigine, gabapentin, topiramate, valproate |
| Lithium | Lithium, lithium carbonate, lithium citrate |
| Anticonvulsants | Acetazolamide, brivaracetam, cannabidiol, clobazam, clonazepam, diazepam, divalproex sodium, eslicarbazepine, ethosuximide, felbamate, fosphenytoin, lacosamide, levetiracetam, magnesium sulfate, methsuximide, oxcarbazepine, perampanel, phenobarbital, phenytoin, pregabalin, primidone, rufinamide, stiripentol, tiagabine, vigabatrin, zonisamide |
| Anxiolytics | Alprazolam, bromazepam, buspirone, chlordiazepoxide, clorazepate, halazepam, hydroxyzine, kava, lorazepam, meprobamate, oxazepam, prazepam, propranolol, diphenhydramine |
| Hypnotics and sedatives | Amobarbital, butabarbital, chlormethiazole, dexmedetomidine, dichloralphenazone, estazolam, eszopiclone, ethchlorvynol, flurazepam, hexobarbital, melatonin, methyprylon, midazolam, nitrazepam, quazepam, ramelteon, scopolamine, suvorexant, tasimelteon, temazepam, triazolam, valerian root extract, zaleplon, zolpidem, zopiclone |
| Stimulants | Amphetamine, armodafinil, atomoxetine, dexmethylphenidate, dextroamphetamine, lisdexamfetamine, methamphetamine, methylphenidate, modafinil, pemoline |
| Substance-abuse drugs | Acamprosate, buprenorphine, disulfiram, methadone, nalmefene, naloxone, naltrexone, nicotine, varenicline |

**Supplementary Table 3: Five most common treatment journeys in the 12-month pre- and post- baseline periods**

| **Medication class at baseline** | **Medication class at 1^st^ switch^a^** | **Proportion of patients, n (%)** |
| --- | --- | --- |
| **12-month period before diagnosis (n=1132)** | | |
| Antidepressants | Antidepressants | 167 (14.8) |
| Anxiolytics + antidepressants | Anxiolytics + antidepressants | 36 (3.2) |
| Antidepressants + anticonvulsants | Antidepressants + anticonvulsants | 32 (2.8) |
| SGA + antidepressants | SGA + antidepressants | 31 (2.7) |
| Antidepressants + mood stabiliser | Antidepressants + mood stabiliser | 28 (2.5) |
| **12-month period after diagnosis (n=1461)** | | |
| Antidepressants | Antidepressants | 128 (8.8) |
| SGA + antidepressants | SGA + antidepressants | 44 (3.0) |
| Anxiolytics + antidepressants | Anxiolytics + antidepressants | 42 (2.9) |
| Antidepressants + mood stabiliser | Antidepressants + mood stabiliser | 29 (2.0) |
| Antidepressants + anticonvulsants | Antidepressants + anticonvulsants | 27 (1.8) |

^a^In cases where patients did not switch, they continued both with the medication class initially prescribed, and the individual medications prescribed within those classes.
SGA, second generation antipsychotic.

**Supplementary Table 4: Most common medication class journeys for patients with BPD in the 12-month period before diagnosis**

| **Medication class at diagnosis** | **Medication class at 1^st^ switch^a^** | **Proportion of patients, n (%)** |
| --- | --- | --- |
| **Antidepressants (n=861)** | | |
| Fluoxetine | Fluoxetine | 72 (8.4) |
| Sertraline | Sertraline | 59 (6.9) |
| Citalopram | Citalopram | 56 (6.5) |
| Escitalopram | Escitalopram | 52 (6.0) |
| Venlafaxine | Venlafaxine | 51 (5.9) |
| **Second-generation antipsychotics (n=405)** | | |
| Quetiapine | Quetiapine | 105 (25.9) |
| Aripiprazole | Aripiprazole | 90 (22.2) |
| Quetiapine | Discontinued | 34 (8.4) |
| Aripiprazole | Discontinued | 29 (7.2) |
| Risperidone | Risperidone | 24 (5.9) |
| **Mood stabilisers (n=307)** | | |
| Lamotrigine | Lamotrigine | 94 (30.6) |
| Gabapentin | Gabapentin | 60 (19.5) |
| Valproate | Valproate | 33 (10.7) |
| Topiramate | Topiramate | 20 (6.5) |
| Gabapentin | Discontinued | 19 (6.2) |

^a^In cases where patients did not switch, they continued both with the medication class initially prescribed, and the individual medications prescribed within those classes.
BPD, borderline personality disorder.

**Supplementary Table 5: Most common medication combinations at baseline stratified by age group**

| **Age Group** | | | | | | | | | | | | | |
| --- | --- | --- | --- | --- | --- | --- | --- | --- | --- | --- | --- | --- | --- |
|  | 12-17 (n=87) |  | 18-25 (n=301) |  | 26-35 (n=415) |  | 36-45 (n=315) |  | 46-55 (n=247) |  | 56-65 (n=82) |  | >65 (n=14) |
| **Number of patients with more than 1 medication prescribed, n (%)** | | | | | | | | | | | | | |
|  | 60 (69.0%) |  | 230 (76.4%) |  | 347 (83.6%) |  | 266 (84.4%) |  | 225 (91.1%) |  | 74 (90.2%) |  | 12 (85.7%) |
| **Number of patients with top 5 most common first-line first recorded medication combinations, n (%)** | | | | | | | | | | | | | |
| aripiprazole, fluoxetine | 5 (5.7%) | clonazepam, sertraline | 10 (3.3%) | clonazepam, quetiapine | 22 (5.3%) | clonazepam, quetiapine | 18 (5.7%) | clonazepam, quetiapine | 22 (8.9%) | clonazepam, fluoxetine | 6 (7.3%) | aspirin, sertraline | 3 (21.4%) |
| escitalopram, trazodone | 4 (4.6%) | aripiprazole, trazodone | 10 (3.3%) | clonazepam, trazodone | 19 (4.6%) | clonazepam, lamotrigine | 14 (4.4%) | clonazepam, sertraline | 17 (6.9%) | fluoxetine, trazodone | 6 (7.3%) | clonazepam, gabapentin | 3 (21.4%) |
| fluoxetine, lamotrigine | 4 (4.6%) | clonazepam, lamotrigine | 9 (3.0%) | citalopram, clonazepam | 17 (4.1%) | aripiprazole, trazodone | 14 (4.4%) | clonazepam, trazodone | 15 (6.1%) | alprazolam, lamotrigine | 5 (6.1%) | bupropion, trazodone | 2 (14.3%) |
| aripiprazole, citalopram | 4 (4.6%) | gabapentin, quetiapine | 9 (3.0%) | clonazepam, sertraline | 15 (3.6%) | quetiapine, zolpidem | 14 (4.4%) | clonazepam, zolpidem | 15 (6.1%) | aripiprazole, trazodone | 5 (6.1%) | clonazepam, venlafaxine | 2 (14.3%) |
| aripiprazole, lamotrigine | 3 (3.4%) | fluoxetine, trazodone | 9 (3.0%) | aripiprazole, clonazepam | 15 (3.6%) | citalopram, clonazepam | 12 (3.8%) | clonazepam, gabapentin | 12 (4.9%) | lamotrigine, quetiapine | 5 (6.1%) | clonazepam, zolpidem | 2 (14.3%) |

Note: A patient may be present in more than 1 combination category. Results are presented as N and % of total number of patients in each age group with ≥12 months of treatment data.

### Supplementary Table 6: Polypharmacy rates at baseline for patients with BPD and comorbid psychiatric disorders

|  | **Comorbid psychiatric disorders with BPD** | | | | | | | | |
| --- | --- | --- | --- | --- | --- | --- | --- | --- | --- |
|  | **MDD** | **PTSD** | **SUD** | **Other bipolar disorder** | **Other mood disorder** | **Anxiety disorder** | **Eating disorder** | **OCD** | **ADHD** |
| **Number of patients, n** | 849 | 486 | 458 | 468 | 328 | 517 | 103 | 68 | 132 |
| **Number of patients with ˃1 medication prescribed, n (%)** | 705 (83.0) | 416 (85.6) | 389 (84.9) | 399 (85.3) | 257 (78.4) | 432 (83.6) | 81 (78.6) | 62 (91.2) | 114 (86.4) |

ADHD, attention deficit hyperactivity disorder; BPD, borderline personality disorder; MDD, major depressive disorder; OCD, obsessive compulsive disorder; PTSD, post-traumatic stress disorder; SUD, substance use disorder.
